# Epidermal growth factor induces a trophectoderm lineage transcriptome resembling that of human embryos during reconstruction of blastoids from extended pluripotent stem cells

**DOI:** 10.1111/cpr.13317

**Published:** 2022-07-26

**Authors:** Yingying Zhang, Chenrui An, Yanhong Yu, Jiajing Lin, Long Jin, Chaohui Li, Tao Tan, Yang Yu, Yong Fan

**Affiliations:** ^1^ Department of Obstetrics and Gynecology, Key Laboratory for Major Obstetric Diseases of Guangdong Province, Key Laboratory of Reproduction and Genetics of Guangdong Higher Education Institutes The Third Affiliated Hospital of Guangzhou Medical University Guangzhou Guangdong China; ^2^ Yunnan Key Laboratory of Primate Biomedical Research, Institute of Primate Translational Medicine Kunming University of Science and Technology Kunming China; ^3^ Beijing Key Laboratory of Reproductive Endocrinology and Assisted Reproductive Technology and Key Laboratory of Assisted Reproduction, Ministry of Education, Center for Reproductive Medicine, Department of Obstetrics and Gynecology Peking University Third Hospital Beijing China

## Abstract

**Objectives:**

This study aims to optimize the human extended pluripotent stem cell (EPSC) to trophectoderm (TE)‐like cell induction with addition of EGF and improve the quality of the reconstructing blastoids.

**Materials and Methods:**

TE‐like cells were differentiated from human EPSCs. RNA‐seq data analysis was performed to compare with TE‐like cells from multiple human pluripotent stem cells (hPSCs) and embryos. A small‐scale compound selection was performed for optimizing the TE‐like cell induction and the efficiency was characterized using TE‐lineage markers expression by immunofluorescence stanning. Blastoids were generated by using the optimized TE‐like cells and the undifferentiated human EPSCs through three‐dimensional culture system. Single‐cell RNA sequencing was performed to investigate the lineage segregation of the optimized blastoids to human blastocysts.

**Results:**

TE‐like cells derived from human EPSCs exhibited similar transcriptome with TE cells from embryos. Additionally, TE‐like cells from multiple naive hPSCs exhibited heterogeneous gene expression patterns and signalling pathways because of the incomplete silencing of naive‐specific genes and loss of imprinting. Furthermore, with the addition of EGF, TE‐like cells derived from human EPSCs enhanced the TE lineage‐related signalling pathways and exhibited more similar transcriptome to human embryos. Through resembling with undifferentiated human EPSCs, we elevated the quality and efficiency of reconstructing blastoids and separated more lineage cells with precise temporal and spatial expression, especially the PE lineage.

**Conclusion:**

Addition of EGF enhanced TE lineage differentiation and human blastoids reconstruction. The optimized blastoids could be used as a blastocyst model for simulating early embryonic development.

## Graphical abstract

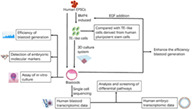

Trophectoderm (TE)‐like cells derived from human extended pluripotent stem cells (EPSCs) form human preimplantation TE by precisely upregulating the expression of multiple TE‐specific marker genes and maintaining gene imprinting. Addition of epidermal growth factor (EGF) promoted TE lineage‐related signalling pathways and increased the efficiency of blastoid generation. Single‐cell transcriptomics showed enhanced lineage segregation of the optimized blastoids to human blastocysts, especially the primitive endoderm, with high fidelity.

## 1 INTRODUCTION

Mammalian embryogenesis starts with a totipotent zygote that can develop into a blastocyst containing epiblast (EPI), primitive endoderm (PE) and trophectoderm (TE).^1^ The establishment of blastocyst models would address the problem of studying natural embryonic development in vivo and the limited available embryos in vitro. Mouse blastocyst‐like structures termed blastoids were formed using mouse pluripotent stem cells (mPSCs).^2^, ^3^ Subsequently, multiple approaches were designed to generate human blastoids using human pluripotent stem cells (hPSCs).^4^, ^5^, ^6^, ^7^, ^8^ These human blastoids separated cell populations specifically expressing some of the EPI, PE and TE lineage makers and exhibited similar functions to human blastocysts. However, cell populations incorrectly expressing lineage markers were observed during reconstruction, as described in previous works,^4^, ^8^ suggesting that these human blastoids still cannot fully represent human blastocysts at the transcriptomic and epigenetic levels. Therefore, a functional blastocyst model must be established to accurately summarize the cell organization and lineage composition of natural human blastocysts.

The establishment of TE plays an important role during the process of generating human blastoids. In natural human embryonic development, TE appears in the blastocyst stage, mediating the interaction at the foetal–maternal interface, and subsequently differentiates into cytotrophoblasts (CTs), syncytiotrophoblasts (STs) and extravillous trophoblasts (EVTs) as implantation occurs.^9^, ^10^, ^11^ Impaired trophectoderm is thought to cause various miscarriages, preeclampsia^12^ and intrauterine growth restriction.^13^, ^14^ Similar to reconstructing human blastoids, using hPSCs is a convenient way to study TE lineage development. The primed hPSCs were considered to be unable to induce functional TE‐like cells^15^, ^16^, ^17^, ^18^ due to the primed pluripotency representing the postimplantation epiblasts.^1^, ^19^ Recently, efforts have been made to develop approaches for resetting naive hPSCs,^20^, ^21^, ^22^, ^23^, ^24^, ^25^ which result in similar features to those of human preimplantation embryos at the transcriptomic and epigenetic levels.^26^, ^27^, ^28^ TE‐like cells derived from naive hPSCs exhibited comparable gene expression patterns to TE cells and successfully differentiated into TE lineage derivatives (CTs, EVTs and STs).^9^, ^29^, ^30^, ^31^ However, high demethylation and loss of imprinting generally occur during naive resetting,^32^ which may disturb embryogenesis and placental development. It is worth studying the feasibility of TE‐like cell induction or reconstruction of human blastoids using naive hPSCs. Human EPSCs are able to form endo‐ or extraembryonic tissues and exhibit higher chimeric efficiency than naive and primed hPSCs.^33^, ^34^, ^35^ In our previous work, we differentiated human EPSCs into TE‐like cells with morphological and transcriptional features similar to those of TE cells. Through assembly of undifferentiated human EPSCs, human blastocyst‐like blastoids were formed with three separated cell lineages and exhibited a transcriptome similar to that of human blastocysts.^8^ In 2018, Okae et al. reported conditions for deriving human trophoblast stem cells (hTSCs), which are capable of transforming into TE lineage derivatives but still require isolation from the human blastocyst or early placenta.^11^

Here, we assessed TE‐like cells derived from different hPSCs and hTSCs with their derivatives. We showed that TE‐like cells derived from human EPSCs were similar to hTSCs and TE from pre‐ or peri‐implantation embryos at the transcriptome level. However, TE‐like cells derived from naive hPSCs exhibited heterogeneous gene expression patterns under different batches or resetting methods. We further found that this heterogeneity was due to the incomplete silencing of naive‐specific genes and loss of imprinting. Upon small molecule compound selection, TE‐like cells derived from human EPSC induction were optimized with the addition of epidermal growth factor (EGF). With undifferentiated human EPSCs, we elevated the quality of reconstructed blastoids and separated more lineage cells with precise temporal and spatial expression, especially the PE lineage. The optimized blastoids showed high similarity to human blastocysts and could be used as a blastocyst model for simulating early embryonic development.

## 2 MATERIALS AND METHODS

### 2.1 ETHICS STATEMENT

This research was performed under the oversight and approval of the Clinical Research Ethics Committee of the Third Affiliated Hospital of Guangzhou Medical University. All procedures were approved by the Institutional Review Board of the Third Affiliated Hospital of Guangzhou Medical University (2020027).

### 2.2 Establishment and culture conditions of human EPSCs

Human EPSCs were cultured in serum‐free N2B27‐LCDM medium under 5% CO_2_ at 37°C and saturated humidity. The method and materials for the establishment and culture conditions of the human EPSCs were described previously.^8^, ^33^, ^36^

### 2.3 Gradient induction experiments with different concentrations of BMP4

The cells were cultured at 37°C, 5% CO_2_ and saturated humidity. Human EPSCs were differentiated with bone morphogenetic protein 4 (BMP4, Catalogue #314‐BP‐010, R&D Systems, CA) for several days. On the starting day, which we called Day 0, the cultured human EPSCs were digested into single cells by TrypLE Express (Catalogue #12604021, Gibco, CA), centrifuged and placed on 0.5% gelatin twice for 20 min each time to remove the feeder. Human EPSCs were seeded in plates pretreated with 1% Matrigel (Catalogue #354277, Corning, NY), which was diluted in DMEM/F12, and the walls were treated for at least 60 min in advance in BMP4 differentiation medium, which was described in our previous work.^8^

### 2.4 Different optimization schemes of BMP4 differentiation

The cells were cultured at 37°C, 5% CO_2_ and saturated humidity. The method of human EPSCs differentiated with BMP4 is as above. In this experiment, the medium of Group BMP4 was composed of 25 ng/mL BMP4 differentiation medium. The medium of the BMP4 + EGF group was composed of 25 ng/mL BMP4 differentiation medium and 50 ng/mL human EGF (Catalogue #EGF L7, Peprotech, NJ). The medium of the BMP4 + VPA group was composed of 25 ng/mL BMP4 differentiation medium and 0.8 mM valproic acid (VPA, Catalogue #227‐01071, Wako, Japan). The medium of the BMP4 + EGF + VPA group was composed of 25 ng/mL BMP4 differentiation medium, 50 ng/mL human EGF and 0.8 mM VPA. Components of the medium of Group HTSC were described previously.^11^ The medium of the HTSC + BMP4 group was composed of HTSC medium with 25 ng/mL BMP4. The medium of Group HTSC − VPA was composed of HTSC medium without VPA. The medium of the HTSC – VPA + BMP4 group was composed of HTSC + BMP4 medium without VPA.

### 2.5 CCK‐8 assay

Cell proliferation was assessed with the Cell Counting Kit‐8 (CCK‐8) assay (Catalogue #40203ES76, Yeasen, CHN). The method of human EPSCs differentiated with different optimization schemes of BMP4 differentiation is as above. The cells were cultured at 37°C, 5% CO_2_ and saturated humidity. Human EPSCs were seeded in 96‐well plates at 2500 cells per well. CCK‐8 solution (10 μl per well) with 90 μl of N2B27 medium was added every 24 h until 120 h, followed by further incubation for 2 h at 37°C. Then, the optical density (OD) was measured at 450 nm.

### 2.6 Generation of blastoids derived from human EPCs in vitro for three‐dimensional culture

The cultured human EPSCs were digested into single cells by TrypLE Express and placed twice on 0.5% gelatin for 20 min each time to remove the feeder. The three‐day BMP4‐differentiated cells were digested into single cells by 0.01% trypsin–EDTA (Catalogue #25300062, Gibco, CA) for 4 min. Human EPSCs (1.0 × 10^5^ cells) and BMP4‐treated cells (5.0 × 10^5^ cells) were mixed together to a total of 6.0 × 10^5^ cells per well with 2.0 ml of blastoid medium and then seeded into one well of a 6‐well aggreWell400 (Catalogue #34425, Stem Cell Technologies, CA) culture plate pretreated with anti‐adherence rinsing solution (Catalogue #07010, Stem Cell Technologies, CA). The blastoid medium was slightly changed every day, and aggregates were collected on the sixth day. The blastoid medium was previously described in our work.^8^

### 2.7 Extended culture of human EPSC‐derived blastoids for 8 and 10 days in vitro

The method and culture conditions of the in vitro cultured embryos were described in previous studies.^8^, ^37^

### 2.8 Immunofluorescence stanning

Dulbecco's phosphate buffered saline (DPBS, Catalogue #C14190500BT, Gibco, MA)‐rinsed samples were fixed with 4% paraformaldehyde for 40 min at room temperature, washed three times with DPBS, and permeabilized with 0.5% Triton X‐100 (Catalogue # 9036‐19‐5, Sigma–Aldrich, MO) in DPBS for 40 min at room temperature. Then, 2% BSA in DPBS was used as blocking buffer, and the samples were blocked with primary antibody diluted in blocking buffer overnight at 4°C. The samples were washed with DPBS containing 0.1% BSA three times. Then, the samples were incubated with fluorescence‐conjugated secondary antibodies diluted in blocking buffer at room temperature for 2 h. Nuclei were stained with 2‐(4‐amidinophenyl)‐6‐indolecarbamidine dihydrochloride (Catalogue #P36941, Invitrogen, CA) at 1 μg/mL. A Nikon immunofluorescence microscope (Nikon A1 R, Tokyo, Japan) was used to capture images. Images were processed by NIS‐Elements Viewer and Fiji (ImageJ, V2.0.0) software. The primary antibodies were as follows: mouse anti‐OCT4 (Catalogue #sc5279, Santa Cruz, CA), mouse anti‐SOX2 (Catalogue #ab171380, Abcam, MA), rabbit anti‐GATA2/3 (Catalogue #ab182747, Abcam, MA), rabbit anti‐TFAP2C (Catalogue #ab76007, Abcam, MA), rabbit anti‐CK8 (Catalogue #ab53280, Abcam, MA), rabbit anti‐CDX2 (Catalogue #ab76541, Abcam, MA), and Alexa Fluor 647 anti‐cytokeratin 7 antibody (Catalogue #ab192077, Abcam, MA). The secondary antibodies were Alexa Fluor 488 goat anti‐rabbit IgG (H + L) (Catalogue # A‐11008, Invitrogen, CA) and Alexa Fluor 594 goat anti‐mouse IgG (H + L) (Catalogue# 8890S, Cell Signaling Technology, MA).

### 2.9 RNA‐seq library preparation and data analysis

Total RNA was isolated using TRIzol. Sequencing was performed on an Illumina X Ten sequencer with a 150 bp paired‐end sequencing reaction. The scRNA sequencing datasets of human embryos were downloaded from ArrayExpress E‐MTAB‐3929^38^ and GSE109555.^39^ The RNA sequencing datasets of TE‐like cells derived from naive and primed hPSCs and their derivatives were downloaded from GSE144994,^29^ GSE167089,^9^ GSE150616^30^ and GSE138762.^31^ All the bulk RNA‐seq data analyses were performed with HISAT2 and Cufflinks using the UCSC human genome annotation (version hg19) with default settings for reads mapping and statistical analysis. The batch effects among the multiple datasets were removed using limma in R. Reads with unique genome location and genes with no less than 1 TPM in at least one sample were used for following analysis. Principal component analysis (PCA) and heatmap analysis were performed with the functions prcomp and pheatmap in R. TPM was normalized by log2 transformation, and the parameter scale was used in the pheatmap and pcromp functions. The plot3D function was used to show the PCA results.

### 2.10 Single‐cell RNA‐sequencing library preparation and data analysis

Day 6 BMP4 + EGF blastoids were collected and prepared to establish an scRNA sequencing library, as described in our previous work.^8^ A previously published single‐cell dataset from Fan et al.^8^ was integrated with the Day 6 BMP4 + EGF blastoid dataset using Find Integration Anchors and Integrate Data. UMAP was used for dimensionality reduction, and FindClusters was used to identify clusters (resolution 0.8). The R package Seurat 4.0.3 was used to analyse the feature‐barcode matrix. DEGs (differentially expressed genes) between lineages were defined with uncorrected *P* values smaller than 0.01 and log‐fold change larger than 0.25 (log2FC > 0.25) in one group.

### 2.11 Statistical analysis

Statistical analyses were performed with GraphPad Prism 8 software using unpaired two‐tailed Student's *t* tests and one‐way ANOVA. All of the statistical tests performed are indicated in the figure legends. The data are presented as the mean ± SD, and *P* < 0.05 was regarded as a significant difference. The significant differences in cell numbers and gene expression between the two samples were analysed by the GraphPad Prism 8 software.

## 3 RESULTS

### 3.1 TE‐like cells derived from human EPSCs are similar to the human trophectoderm lineage at the transcriptomic level

We converted human induced pluripotent stem cells (hiPSCs) into human EPSCs by an established protocol.^8^, ^33^, ^36^ The obtained human EPSCs exhibited a dome shape (Figure S1A), high alkaline phosphatase activity (Figure S1B), a normal karyotype (Figure S1C) and high expression of pluripotent cell markers (Figure S1D). Teratoma assays verified the ability to differentiate into three germ layers (Figure S1E).

**FIGURE 1:**
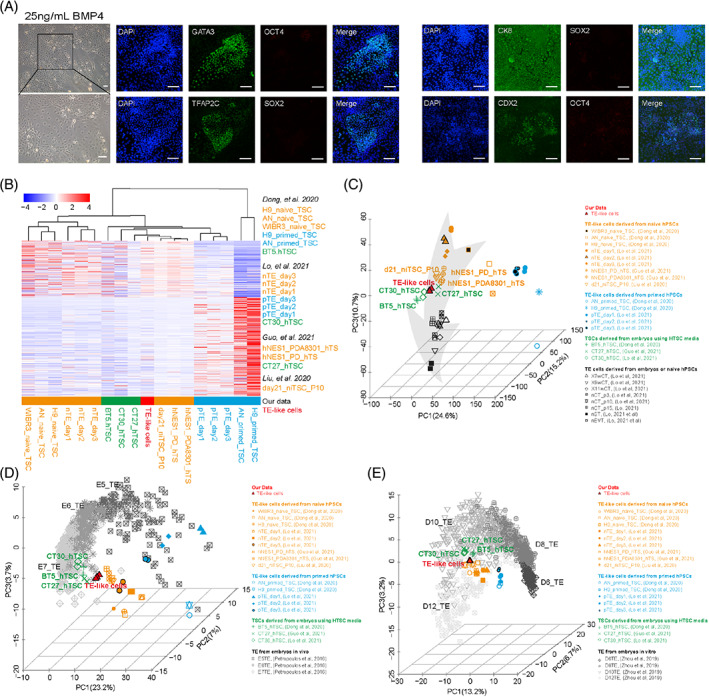
Characterization of TE‐like cells derived from human EPSCs, naive hPSCs, primed hPSCs and hTSCs compared with TE at the transcriptome level. (A) Left panel: representative images of TE‐like cells induced from human EPSCs using 25 ng/mL BMP4. Right panel: representative images of TE‐like cells, detecting the expression of TE lineage‐specific markers (*GATA3*, *TFAP2C*, *CK8*, *CDX2*) and pluripotent markers (*OCT4*, *SOX2*) by immunofluorescence staining. All images were taken at Day 3 during induction. Scale bars indicate 100 μm. (B) Heatmap of RNA‐seq data from TE‐like cells derived from human EPSCs (red, this study), naive hPSCs (orange^9^, ^29^, ^30^, ^31^), primed hPSCs (blue^29^, ^31^) and hTSCs (green^9^, ^29^, ^31^). Hierarchical clustering was based on the genes with a fold change greater than 1.5 between all TE‐like cells derived from naive and primed hPSCs TPM. (C) PCA of RNA‐seq data from TE‐like cells derived from human EPSCs, naive hPSCs, primed hPSCs and hTSCs derived from embryos and their derivatives (black^29^). Each single symbol represents one sample in each cell line. (D) PCA of RNA‐seq data compared with single‐cell RNA‐seq data from human early embryos in vivo.^38^ Each single symbol represents one sample in each cell line or one cell in human early embryos at different embryonic days. (E) PCA of RNA‐seq data compared with single‐cell RNA‐seq data from human early embryos in vitro.^39^ Each single symbol represents one sample in each cell line or one cell in human early embryos at different embryonic days.

According to previous work,^8^ we differentiated human EPSCs into TE‐like cells under 25 ng/mL BMP4 induction (Figure 1A). Three days later, the TE‐like cells derived from human EPSCs showed specifically elevated gene expression levels of TE lineage markers (*GATA3*, *TFAP2C*, *CK8*, *CDX2*),^40^, ^41^, ^42^, ^43^ accompanied by downregulated expression of pluripotent markers (*OCT4*, *SOX2*) (Figure 1A). We next performed RNA sequencing (RNA‐seq) of the TE‐like cells derived from human EPSCs to compare multiple datasets of TE‐like cells derived from naive hPSCs,^9^, ^29^, ^30^, ^31^ primed hPSCs^29^, ^31^ and hTSCs from embryos.^9^, ^29^, ^31^ Hierarchical clustering analysis effectively separated each cell type and showed that TE‐like cells derived from human EPSCs clustered together with hTSCs and that all TE‐like cells derived from naive hPSCs displayed similar gene expression patterns (Figure 1B), while the TE‐like cells derived from primed hPSCs differed from the other cells (Figure 1B). Principal component analysis (PCA) gave consistent results in comparisons of TE‐like cells derived from human EPSCs, naive hPSCs, primed hPSCs, hTSCs and their derivatives (CTs, STs and EVTs) (Figure 1C).

To further distinguish the TE‐like cells derived from multiple hPSCs, we performed PCA with comparison between the RNA‐seq data used above and the single‐cell RNA‐seq (scRNA‐seq) data of pre‐^38^ or peri‐implantation^39^ human embryos. We found that the TE‐like cells from human EPSCs (this study), 2iLGöY^30^ and PXGL^9^ naive hPSCs and hTSCs were closer to the E7 TE of preimplantation embryos, while TE‐like cells derived from 2iLGö^29^ and 5iLA^31^ naive hPSCs and primed hPSCs were positioned away (Figure 1D). Compared to the TE of peri‐implantation embryos, the TE‐like cells from human EPSCs (this study), 5iLA^31^ naive hPSCs and hTSCs gathered together approaching Day 10 (a postimplantation blastocyst stage) TE cells (Figure 1E). However, the TE‐like cells from naive hPSCs, similar to preimplantation TE cells, seemed to separate together with those cells from primed hPSCs (Figure 1E). These results suggested that human EPSC‐derived TE‐like cells shared a transcriptome similar to that of pre‐ and peri‐implantation embryos and captured both gene expression patterns in pre‐ and postimplantation embryos, which may be due to the intermediate pluripotency of human EPSCs between naive and primed hPSCs. In contrast, TE‐like cells derived from different research groups or different naive hPSCs exhibited a heterogeneous transcriptome, and TE‐like cells differentiated from primed hPSCs could not mimic the embryonic trophectoderm lineage.

### 3.2 TE‐like cells derived from human EPSCs, but not from naive hPSCs, correctly enhance TE signalling pathways and maintain imprinting

**FIGURE 2:**
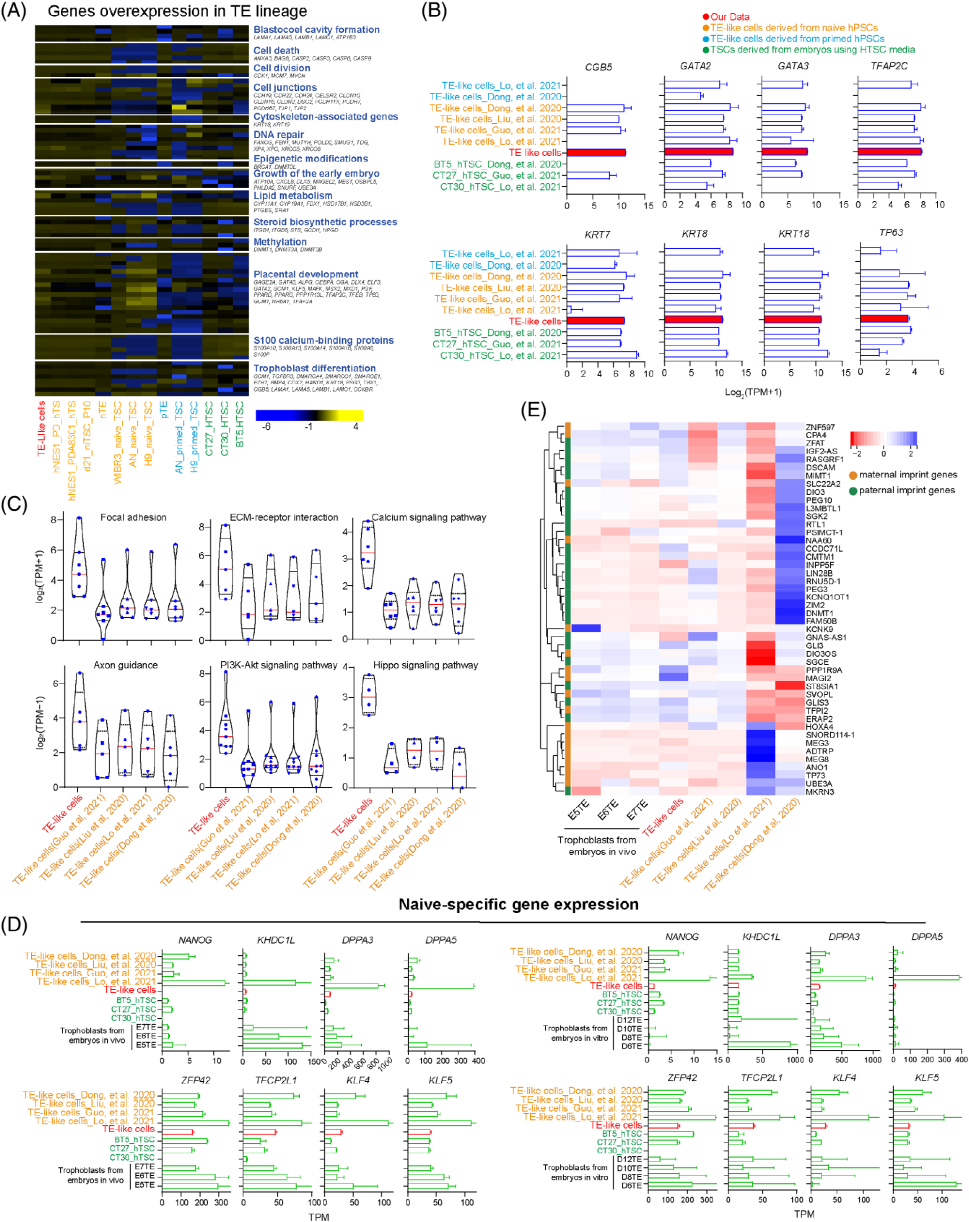
TE‐like cells derived from human EPSCs enhance TE‐specific signal pathways, while those from naive hPSCs exhibit incomplete pluripotency and lose imprinting. (A) Heatmap of the expression patterns of selected TE lineage‐enhanced genes in TE‐like cells derived from human EPSCs, naive hPSCs, primed hPSCs and hTSCs. (B) Expression of TE‐specific genes in TE‐like cells derived from human EPSCs, naive hPSCs, primed hPSCs and hTSCs. The expression levels of genes are represented using Log2(TPM + 1). Error bars represent the SD of replicates. (C) Expression of differentially expressed pathway‐related genes in TE‐like cells derived from human EPSCs and naive hPSCs. The expression levels of genes are represented using the average of replicates or single cells' Log2(TPM + 1). Each blue dot represents one gene. Red lines in each column represent medians of expression among all genes. (D) Expression of naive‐specific genes in TE‐like cells derived from human EPSCs, naive hPSCs, and TE in vivo (left panel) and in vitro (right panel). The expression levels of genes are represented using Log2(TPM + 1). Error bars represent the SD of replicates. (E) Heatmap showing the expression patterns of selected imprinted genes (based on the Geneimprint database, https://www.geneimprint.com/site/genes‐by‐species) in TE‐like cells derived from human EPSCs, naive hPSCs, and TE in vivo.

To further characterize TE‐like cells derived from human EPSCs, naive hPSCs, primed hPSCs and hTSCs, we analysed the expression of all genes with upregulated expression in the TE lineage.^44^ The expression levels of TE‐like cells derived from human EPSCs and 2iLGöY^30^ and PXGL^9^ naive hPSCs were much higher than those of other cells, even compared with those of hTSCs (Figure 2A). However, all cells consistently expressed TE lineage‐specific genes (*CGB5*, *GATA2*, *GATA3*, *TFAP2C*, *KRT7*/*8*/*18* and *TP63*) at similar levels (Figure 2B), which was also found in the comparison of gene expression related to placental development, yolk sac formation and amniotic membrane or fluid (Figures S2A, S2B and S2C). This finding suggests that there are some non‐TE lineages directly related to factors in TE‐like cells derived from naive hPSCs causing the occurrence of heterogeneous gene expression patterns.

Next, we compared the DEGs between TE‐like cells derived from human EPSCs and naive hPSCs (Figure S2D). KEGG analysis showed that genes with upregulated expression in the TE‐like cells derived from human EPSCs were enriched in multiple TE‐related signalling pathways based on previous studies, including focal adhesion,^45^ the PI3K‐Akt signalling pathway,^46^ extracellular matrix (ECM)‐receptor interactions,^47^ the TGFβ signalling pathway,^48^, ^49^ the calcium signalling pathway,^50^ axon guidance and the Hippo signalling pathway,^16^ compared with the TE‐like cells derived from naive hPSCs and primed hPSCs (Figures 2C and S2E). Among them, the ECM signalling pathway plays a major role in TE development.^47^, ^51^ We selected multiple genes related to ECM and TE, including *COL1A1*, *KDR*, *RELN*, *ITGB6*, *VEGF*C, *FGF10*, *GDF7*, *TGFB2* and *TGFB3*.^38^, ^48^, ^49^, ^52^, ^53^, ^54^, ^55^, ^56^, ^57^ Indeed, the expression levels of these genes in the TE‐like cells derived from human EPSCs were significantly higher than those in the TE‐like cells derived from naive hPSCs (Figure S2F). Interestingly, both the TE‐like cells derived from naive and primed hPSCs upregulated the wingless/integrated (WNT) signalling pathway (Figure S2G) compared with those from human EPSCs, which is considered to be essential for hTSCs in previous work.^11^ These results indicate that the heterogeneous enhancement of TE‐related signalling pathways is the reason why TE‐like cells derived from naive and primed hPSCs partially obtain TE features.

More importantly, we found that TE‐like cells derived from naive hPSCs significantly elevated the signalling pathway regulating pluripotency of stem cells in comparison with those from human EPSCs (Figure S2G). To further explore the effect of pluripotency on TE differentiation, we detected the expression of naive‐specific genes and observed higher expression levels in the TE‐like cells derived from naive hPSCs than the others (Figure 2D). This finding indicates that naive hPSCs did not completely exit naive pluripotency during TE‐like cell induction. Additionally, owing to genome‐wide demethylation and loss of imprinting^27^, ^58^, ^59^, ^60^, ^61^ during naive resetting, we investigated whether these epigenetic features would be maintained in TE‐like cells derived from naive hPSCs. Upon investigation of the expression of imprinted genes, TE‐like cells derived from human EPSCs and 2iLGöY^30^ and PXGL^9^ naive hPSCs showed highly consistent gene expression patterns with TE^38^ cells, in contrast to 2iLGö^29^ and 5iLA^31^ naive hPSCs (Figure 2E), consistent with our abovementioned PCA data. This finding suggested that this heterogeneity of TE‐like cells derived from naive hPSCs was due to the incomplete silencing of naive‐specific genes and loss of imprinting.

### 3.3 TE‐like cells derived from human EPSC induction are optimized under small molecule compound selection

In 2018, Okea et al. established an hTSC culture condition using EGF and WNT activator (CHIR99021), while inhibiting transforming growth factor beta (TGFβ), histone deacetylase (HDAC), and Rho‐associated kinase (ROCK) signalling pathways, called HTSC medium.^11^ HTSCs can differentiate into three main trophoblast cells with molecular characteristics, transcription levels and secretion functions similar to those of placental cells in early pregnancy.

**FIGURE 3:**
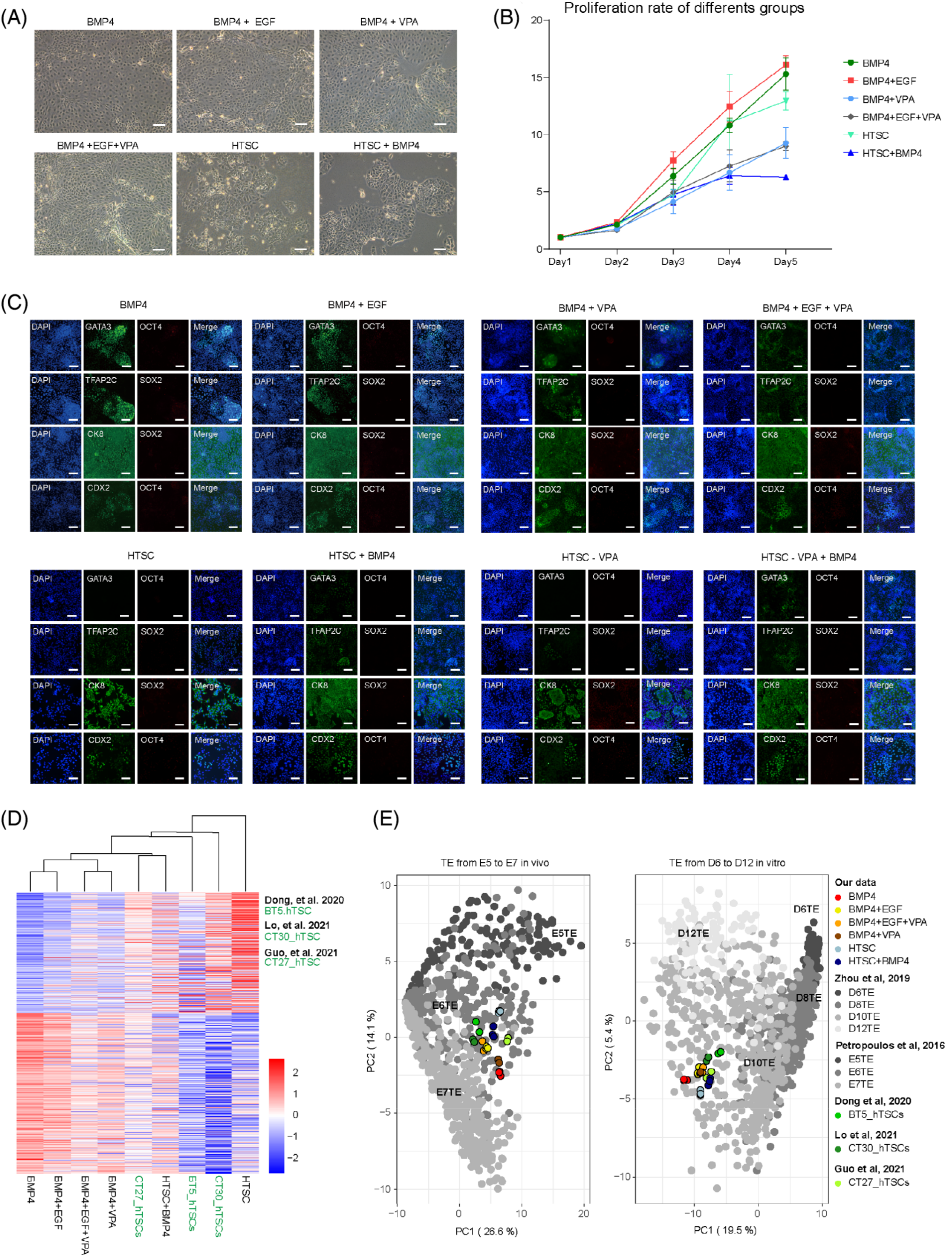
TE‐like cells derived from human EPSC induction are optimized with small molecule compound selection. (A) Representative images of TE‐like cells derived from human EPSCs with induction of six grouped small molecule combinations. Scale bars indicate 100 μm. (B) The curves show the proliferation rate of TE‐like cells induced by multiple groups of small molecule combinations from Days 1 to 5. The *Y*‐axis represents the proliferation rate. (C) Representative images of TE‐like cells induced by different groups of small molecule combinations, detecting the expression of TE lineage markers (*GATA3*, *TFAP2C*, *CK8*, *CDX2*) and pluripotent markers (*OCT4*, *SOX2*) by immunofluorescence staining. All images were taken at Day 3 during induction. Scale bars indicate 100 μm. (D) Heatmap of RNA‐seq data from TE‐like cells under multiple induction conditions. Hierarchical clustering was based on the genes with a fold change greater than 1.5 between TE‐like cells derived using BMP4 and HTSC TPM. (E) PCA of RNA‐seq data compared with single‐cell RNA‐seq data from human early embryos in vivo (left panel^38^) and in vitro (right panel^39^). Each single symbol represents one sample in each cell line or one cell in human early embryos at different embryonic days.

To further optimize the previously established TE‐like cells derived from human EPSCs induced by the BMP4 induction system, we conducted small‐scale compound selection based on HTSC medium. We chose EGF or VPA combined with BMP4 to form six TE‐like cells derived from human EPSC induction programs. Other compounds were excluded because CHIR99021 and Y27632 both existed in the human EPSC LCDM medium,^33^ and A83‐01 is a signalling pathway antagonist of BMP4. After 3 days of induction, all the groups treated with BMP4 showed cobblestone‐shaped cells, while the HTSC medium‐induced TE‐like cells were island‐shaped (Figure 3A). Adding EGF increased the cell proliferation rate in the presence of BMP4, in contrast to the addition of VPA (Figure 3B). Accordingly, we added the HTSC − VPA and HTSC − VPA + BMP4 groups as controls during TE‐like cell induction. The expression levels of *GATA3* in the BMP4‐based TE‐like cells were higher than those in the HTSC groups, while there were no significant differences of the other TE markers among these cells (Figures 3C, S2H and S2I).

Next, we performed RNA‐seq on the TE‐like cells derived from human EPSCs. Hierarchical clustering clearly distinguished the TE‐like cells derived using different induction systems, and the BMP4‐based groups clustered together, while the TE‐like cells induced by HTSC were separated (Figure 3D). We also found that hTSCs from different embryos exhibited heterogeneous gene expression patterns (Figure 3D), suggesting that the HTSC induction system has uneven stability. PCA showed that among the BMP4‐treated groups, the addition of EGF or VPA resulted in more similarity to TE in vivo^38^ or in vitro^39^ at the transcriptome level (Figure 3E). Thus far, we further confirmed that BMP4 has a central role in inducing TE‐like cells derived from human EPSCs and that the addition of EGF further promotes TE‐like cells to become similar to TE cells in vivo or in vitro.

### 3.4 Human blastoid construction is enhanced using TE‐like cells derived from human EPSCs induced by EGF

**FIGURE 4:**
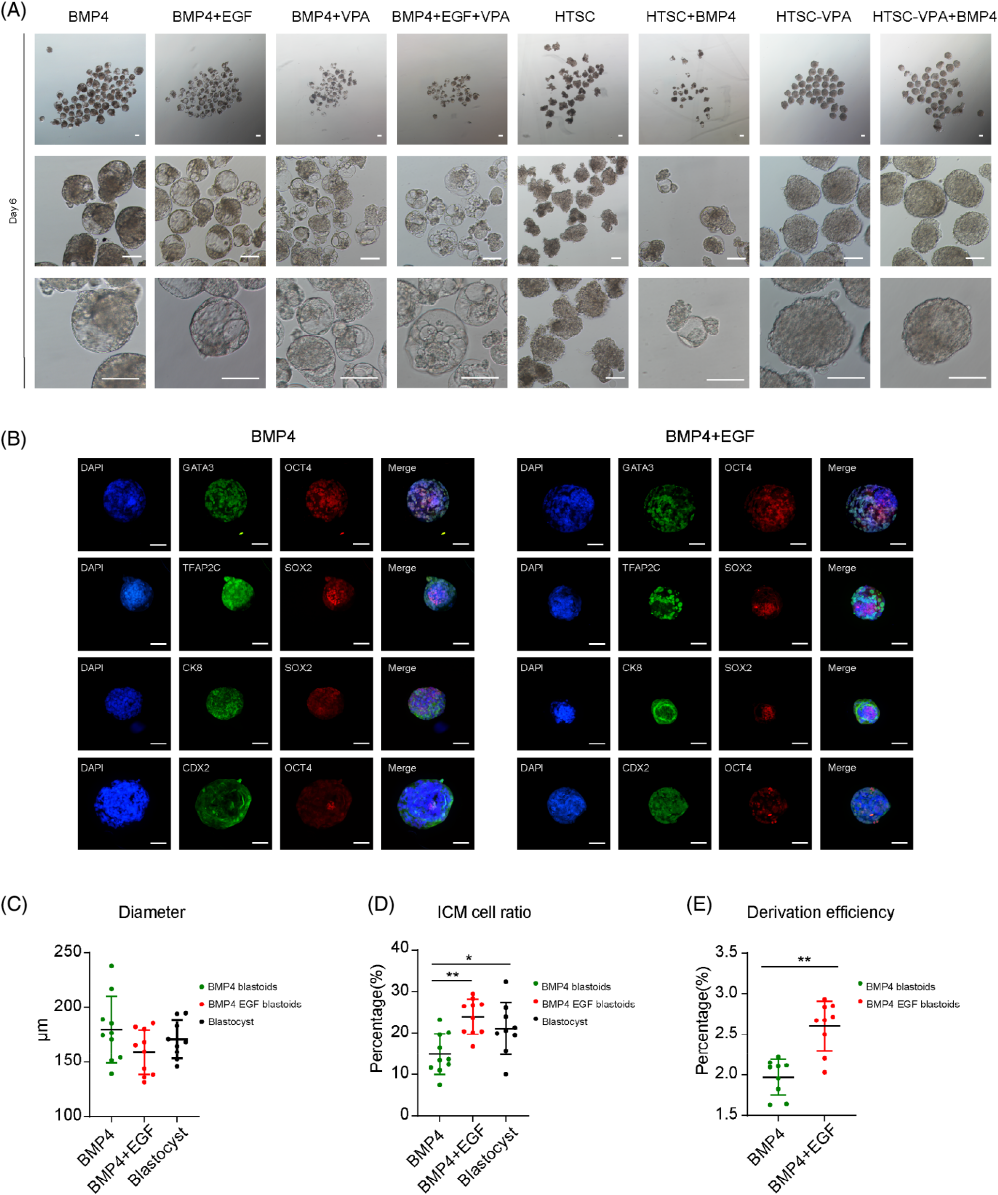
Blastoid reconstruction is enhanced using TE‐like cells induced by adding EGF. (A) Representative images of blastoids using TE‐like cells induced by different grouped small molecule combinations at Day 3 and Day 6. Scale bars indicate 100 μm. (B) Representative images of blastoids reconstructed from TE‐like cells induced by BMP4 and BMP4 + EGF, detecting the expression of TE lineage‐specific markers (*GATA3*, *TFAP2C*, *CDX2*, *CK8*) and pluripotent markers (*OCT4*, *SOX2*) by immunofluorescence staining. All images were taken at Day 6 during induction. Scale bars indicate 100 μm. (C) Diameter was quantified between human blastoids generated from the BMP4 and BMP4 + EGF groups and blastocysts. Data are the mean ± SD (*n* = 10 blastoids). *P < 0.05, **P < 0.01. (D) The ICM cell ratio was quantified between human blastoids generated from the BMP4 and BMP4 + EGF groups and blastocysts, data are mean ± SD (*n* = 10 blastoids). *P < 0.05, **P < 0.01. (E) Derivation efficiency was quantified between human blastoids generated from the BMP4 and BMP4 + EGF groups. Data are the mean ± SD (*n* = 9 times). *P < 0.05, **P < 0.01

To identify the function of TE‐like cells derived from human EPSCs under different induction conditions, we carried out the reconstruction of blastoids according to our previous work. In the three‐dimensional culture system early on the third day, cell aggregates of the BMP4 and BMP4 + EGF groups formed small cavities (Figure 4A). On Day 6, the BMP4 and BMP4 + EGF groups formed obvious blastocyst‐like structures, similar in shape to human blastocysts, including a dense inner cell mass (ICM), a cavity and circular surrounding cells (Figure 4A). Few blastoids were generated from the HTSC + BMP4 and VPA groups with poor shapes (Figure 4A). Blastoids generated from the BMP4 and BMP4 + EGF groups accurately expressed ICM markers (*OCT4*, *SOX2*), TE markers (*GATA3*, *TFAP2C*, *CDX2*, *CK8*, *CK7*) and PE maker (*GATA6*) through immunofluorescence staining and microscoping with or without the *Z* axis (Figures 4B and S3A). However, the cell aggregates derived from other groups incompletely expressed lineage‐specific genes (Figure S3B). In comparison to natural human blastocysts, these human blastoids generated from the BMP4 and BMP4 + EGF groups exhibited similar shapes, including similar average diameters (Figure 4C) and ICM ratios (Figure 4D). In addition to EGF, the efficiency of blastoid generation was 1.5 times higher than that in the BMP4 group (Figures 4E, S4A and S4B), suggesting that the addition of EGF strengthens the establishment of blastoids by optimizing the induction of TE‐like cells.

To further assess the role of EGF in TE‐like cell induction by BMP4, we analysed the DEGs between the TE‐like cells induced by BMP4 and BMP4 + EGF (Figure S4C). KEGG and GO term analyses showed that the BMP4 + EGF‐TE‐like cells further enhanced TE lineage‐related signalling pathways, such as the ECM‐receptor interaction, TGFβ signalling pathway and Hippo signalling pathway, compared with the BMP4‐TE‐like cells (Figure S4D). Moreover, adding EGF enhanced the WNT and pluripotency of stem cell signalling pathways (Figure S4D), which is consistent with previous reports.^11^ Both BMP4 and BMP4 + EGF blastoids collapsed under extended culturing (Figure S4E). Compared to the previous results of generating blastoids using mouse EPSCs independently,^62^ we did not achieve blastocyst‐like structures using human EPSCs alone or TE‐like cells induced with BMP4 treatment (Figure S4F). To identify whether blastoids simulated postimplantation morphogenesis, we used the in vitro culture condition (IVC) of human embryos to extended culturing of BMP4 or BMP4 + EGF blastoids. The morphological characteristics of blastoids on Days 8, 10 and 12 were consistent with previous work,^8^ exhibiting 31.1% and 40% adherence rates (Figure S4G) and correct expression of ICM and TE lineage markers (Figure S4H). In summary, these results indicate that the BMP4 + EGF induction system functionally optimizes the differentiation of TE‐like cells and improves the efficiency of generating human blastoids.

### 3.5 Single‐cell transcriptome analysis of three lineages of blastoids

**FIGURE 5:**
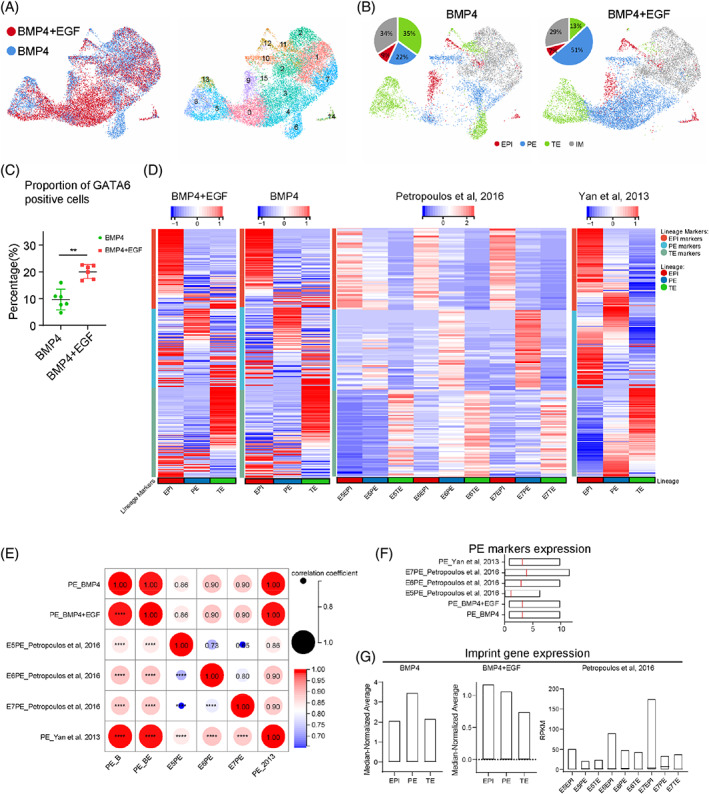
Optimized blastoids enhance PE lineage differentiation and reduce the IM cell population. (A) UMAP plots showing integrated datasets of BMP4 and BMP4 + EGF blastoids (left panel) and were divided into 16 major clusters (right panel). (B) UMAP plots showing EPI, PE, TE and IM cell populations of BMP4 and BMP4 + EGF blastoids, defined using the lineage‐specific markers in the CellMaker database. (C) The proportions of GATA6 positive cells were quantified by counting the cell number using the immunofluorescence stanning images of the blastoids generated from the BMP4 and BMP4 + EGF groups. Data are the mean ± SD (*n* = 6 blastoids). *P < 0.05, **P < 0.01. (D) Heatmap showing gene expression patterns of EPI, PE and TE lineages of BMP4 and BMP4 + EGF blastoids compared to human preimplantation embryos in vivo.^38^, ^63^ (E) Dot plot indicating the gene expression pattern correlation of PE cells from BMP4 and BMP4 + EGF blastoids and human preimplantation embryos in vivo^38^, ^63^ using PE lineage markers. (F) Comparison of PE lineage markers among the BMP4, BMP4 + EGF and human blastocyst groups in vivo.^38^, ^63^ (G) Comparison of imprinted gene expression among the BMP4, BMP4 + EGF and human blastocyst groups in vivo.^38^

In our previous work,^8^ by comparing the single‐cell transcriptome analysis between Day 6 BMP4 human blastoids and E5–E7 human blastocysts,^38^ we proved that the blastoids derived from human EPSCs captured the EPI, TE and PE cell populations that mostly overlapped with the blastocysts. However, many cells express both ICM and TE markers, termed an intermediate (IM) cell population, which cannot represent human embryos. Next, we wondered whether EGF‐optimized blastoids could reduce the intermediate cell population and became closer to human blastocysts. We performed scRNA‐seq of Day 6 BMP4 + EGF blastoids to compare the scRNA‐seq data of BMP4 blastoids from our previous work. Uniform manifold approximation and projection (UMAP) analysis revealed largely overlapping distributions of BMP4 and BMP4 blastoids with 16 divided clusters (Figure 5A). To define these clusters at high resolution, we selected dozens of lineage‐related genes based on the CellMarker database (http://biocc.hrbmu.edu.cn/CellMarker/index.jsp) (Figure S5A). We separated the clusters into EPI, PE and TE cell populations according to the number of lineage markers expressed (Figure 5B). Intermediate (IM) cell populations were also found to express lineage markers incorrectly, consistent with previous work^38^, ^63^ (Figure 5B). Notably, the BMP4 + EGF blastoids had a 5% decrease in the IM cell populations while achieving more PE cells compared to the BMP4 blastoids (Figure 5B). By counting the cell number, the proportion of GATA6 (a PE lineage marker) positive cells from BMP4 + EGF group is 2 times higher than that from BMP4 group (Figure 5C), suggesting the increasing PE cell number. We further verified lineage marker expression compared with that of human preimplantation embryos.^38^, ^63^ The separated cell populations of the BMP4 + EGF and BMP4 blastoids both expressed the most markers in the specific lineages, similar to human preimplantation embryos (Figure 5D). The characterization of multiple well‐defined lineage marker genes showed consistent results (Figure S5B). Next, we wondered whether the increased PE cell population of the BMP4 + EGF blastoids truly resembled the PE lineage. The Pearson correlation coefficient showed that this PE cell population was close to PE cells from E7^38^ and Day 6^63^ human embryos (Figure 5E) with similar PE lineage gene expression levels (Figure 5F). In comparisons of imprinting gene expression patterns in human three embryonic lineages, the expression levels of imprinting genes in the pre‐implantation epiblasts (EPI) were higher than those in TE and PE, no matter at E5, E6 or E7 (Figure 5G). We also found the similar expression patterns in the BMP4 + EGF blastoids but not in the BMP4 blastoids (Figure 5G).

Thus, by optimizing TE‐like cell induction with the addition of EGF, we increased the efficiency of blastoid generation and achieved more cell lineage formation under high‐resolution clustering, especially in the PE lineage.

## 4 DISCUSSION

Trophectoderm formation is the first differentiation event in human embryogenesis. Due to ethical restrictions and few available embryos, TE‐like cell derivation using pluripotent stem cells is the main method to study trophectoderm development. Single‐cell RNA‐seq analysis demonstrated that primed hPSCs are close to the late postimplantation ectoderm when TE and EPI lineages separate,^1^ resulting in TE‐like cells differentiated from primed hPSCs that tend to transform into amniotic cells.^41^ Recently, many efforts have been made to convert naive hPSCs into TE‐like cells with transcriptional features similar to those of embryonic trophoblasts.^9^, ^29^, ^30^, ^31^ Subsequently, blastocyst‐like blastoids were reconstructed using naive hPSCs divided into three main lineages and sharing similar transcriptomes with human embryos.^4^, ^5^, ^6^, ^7^, ^8^ However, IM cell populations were reported in multiple previous works.^4^, ^8^ In the present study, we found that TE‐like cells derived from naive hPSCs did not completely turn off naive‐specific gene expression. This phenomenon may be an important reason for the formation of IM cell populations. Persistently expressed naive‐specific genes contribute to differences in the degree and progression of cell differentiation and organization. Furthermore, these blastoids cannot accurately depict the state of a human embryo at a specific stage. Genomic imprinting is of mammalian parental origin and relies on the monoallelic expression of a set of genes that are essential for embryonic development.^64^, ^65^ The imprinting signature is mostly established in the germline and maintained even if the embryo undergoes extensive demethylation after fertilization but is demethylated only in the new primordial germ cells.^65^ Unlike the preimplantation epiblast, naive hPSCs lose methylation at imprinted differentially methylated regions (iDMRs) and do not regain methylation at these loci upon differentiation.^27^, ^58^ Indeed, we observed heterogeneous gene expression patterns of TE‐like cells derived from naive hPSCs, while TE‐like cells derived from human EPSCs shared a similar imprinting signature to TE. This result indicates that both TE‐like cells and blastoids derived from naive hPSCs have a risk of loss of imprinting, demonstrating a hidden risk for subsequent research on embryonic development.

Human EPSCs have a certain potential for TE differentiation. In this study, a number of signalling pathways related to the TE lineage were significantly enhanced compared with those in the TE‐like cells derived from naive or primed hPSCs. The related genes in these pathways were confirmed to be involved in regulating the development of the TE lineage in multiple previous studies. *COL1A1* is considered a marker for osteogenic differentiation of amniotic membrane cells.^52^ *ITGB6* and *EGFR* are commonly used as cell surface markers for simultaneously labelling CTs and hTSCs. The combination of the TE‐like cell surface marker *ITGB6* and latency‐associated peptides can promote embryo implantation.^56^ *VEGFC* is mainly produced by decidual natural killer cells in the foetal–maternal interface, which can improve immune tolerance and angiogenesis^54^ and induce TE cells to differentiate into EVTs.^55^ TGFβ2/β3 negatively regulate TE invasion and EVT differentiation.^48^, ^49^ Thus, these genes are involved in the differentiation, development and invasion of TE cells by interacting with and influencing each other to form a rigorous signal regulatory network.

In early pregnancy, *EGF* and EGFR are expressed in the trophoblast cells of the placenta and stimulate cell proliferation and hormone production.^66^ The EGF/EGFR and VEGF/VEGF receptor (VEGFR) loops may play a major role among the autocrine and paracrine loops related to TE proliferation.^67^ A previous work reported that *EGF* downregulated *KISS1* expression by activating the *PI3K*/*AKT* signalling pathway to stimulate human TE cell invasion.^46^ *EGF* is necessary for epithelial stem cell proliferation and cooperates with forskolin, an EPAC/RAP1 agonist, to enhance the formation of sac‐like structures.^11^ Additionally, EGFR is commonly used as a surface marker for labeling CTs and hTSCs.^31^, ^53^ Based on these previous works and our findings, we consider that the addition of EGF strengthens TE‐like cell induction by enhancing ECM‐receptor interactions and the *TGFβ*, *WNT* and *PI3K*/*AKT* signalling pathways. From natural human embryos, EPI and TE cells are easier to obtain than PE cells. There is currently no mature way to derive PE cells because of the slower growth than that of EPI and TE cells.^7^ PE markers are usually expressed in TE cells, which makes it difficult to identify the true PE cell population. The addition of EGF during the generation of blastoids increased the proportion of the PE cell population with high similarity to that of human embryos, which offers a robust way to derive PE cells.

Previously, some important regulatory genes have been found in mice, such as *Cdx2* and *Rif1*. Knocking out *Rif1* plays critical roles in the regulation of trophoblast cells in mice.^68^ Notch and Hippo signalling pathways cross talk to activate *Cdx2* expression in mouse preimplantation embryos and promote the specialization of trophectoderm.^69^ Overexpressing *Cdx2* converted haploid ESCs to TSCs,^70^ suggesting *Cdx2* influenced the pluripotency. There is a cross‐regulation of the *Nanog* and *Cdx2* promoters during trophoblast differentiation and *Cdx2* can represses *Oct4* expression.^71^ These indicated that *Cdx2* and *Rif1* are key regulators for specialization of TE lineage cells. To verify the function of these regulators in human TE lineage cells, it is necessary to establish a cell line resembling the TE in vivo. However, the current method of differentiating human TE lineage cells still needs to be optimized. In this study, we found the expression of *Cdx2* in the induced TE‐like cells but not in the blastoids. In the previous work, *Cdx2* is mentioned as a medium TE maker which is temporarily up‐regulated during the preimplantation embryos,^72^ We suspect Cdx2 initiates TE differentiation in the early stage, but fails to sustain in subsequent development.

In summary, by assessing TE‐like cells derived from multiple hPSCs, we demonstrated that TE‐like cells derived from human EPSCs represented the TE of preimplantation embryos and optimized TE‐like cell induction by adding EGF. The application of optimized TE‐like cells effectively improved the efficiency of reconstructing blastoids and provided a robust method to generate PE cells.

## AUTHOR CONTRIBUTIONS

Yingying Zhang and Chenrui An provided direction in experimental design, performed the majority of the experiments, interpreted the data and wrote the manuscript. Yingying Zhang performed the majority of the experiments related to TE differentiation from human EPSCs and their characterizations. Jiajing Lin, Yanhong Yu and Long Jin performed the blastoid collection, immunofluorescence labelling and the blastoid in vitro culture experiments. Chenrui An, Chaohui Li and Tao Tan performed the bioinformatics and data analysis. Yong Fan and Yang Yu provided resources and conceived and supervised the study.

## CONFLICT OF INTEREST

The authors declare that the research was conducted in the absence of any commercial or financial relationships that could be construed as a potential conflict of interest.

## Supporting information


**Figure S1** Characterization of human EPSCs derived from human iPSCs
**Figure S2.** TE‐like cells derived from human EPSCs share a similar gene expression pattern with hTSCs, and those from naive hPSCs did not completely turn off naive‐specific gene expression (related to Figure 2).
**Figure S3.** Human blastoid reconstruction using TE‐like cells derived from human EPSCs induced by multiple small molecule combinations, related to Figure 4.
**Figure S4.** Human blastoid construction is improved by further enhancement of TE‐specific signal pathways by adding EGF, related to Figure 4.
**Figure S5.** Gene expression patterns of three lineages of blastoids generated from human EPSCs, related to Figure 5

## Data Availability

The datasets generated during this study are available at Sequence Read Archive (SRA) with the accession number PRJNA799588.
