# Supplementary material for: Epidermal growth factor induces a trophectoderm lineage transcriptome resembling that of human embryos during reconstruction of blastoids from extended pluripotent stem cells
Source: Cell Prolif. 2022 Jul 26;55(11):e13317. doi: 10.1111/cpr.13317 (PMC9628219; doi:10.1111/cpr.13317)
Supplement: Supplementary file 1 — Figure S1 Characterization of human EPSCs derived from human iPSCs Figure S2. TE‐like cells derived from human EPSCs share a similar gene expression pattern with hTSCs, and those from naive hPSCs did not completely turn off naive‐specific gene expression (related to Figure 2). Figure S3. Human blastoid reconstruction using TE‐like cells derived from human EPSCs induced by multiple small molecule combinations, related to Figure 4. Figure S4. Human blastoid construction is improved by further enhancement of TE‐specific signal pathways by adding EGF, related to Figure 4. Figure S5. Gene expression patterns of three lineages of blastoids generated from human EPSCs, related to Figure 5 [file CPR-55-e13317-s001.doc]

**SUPPLEMENTAL FIGURES**


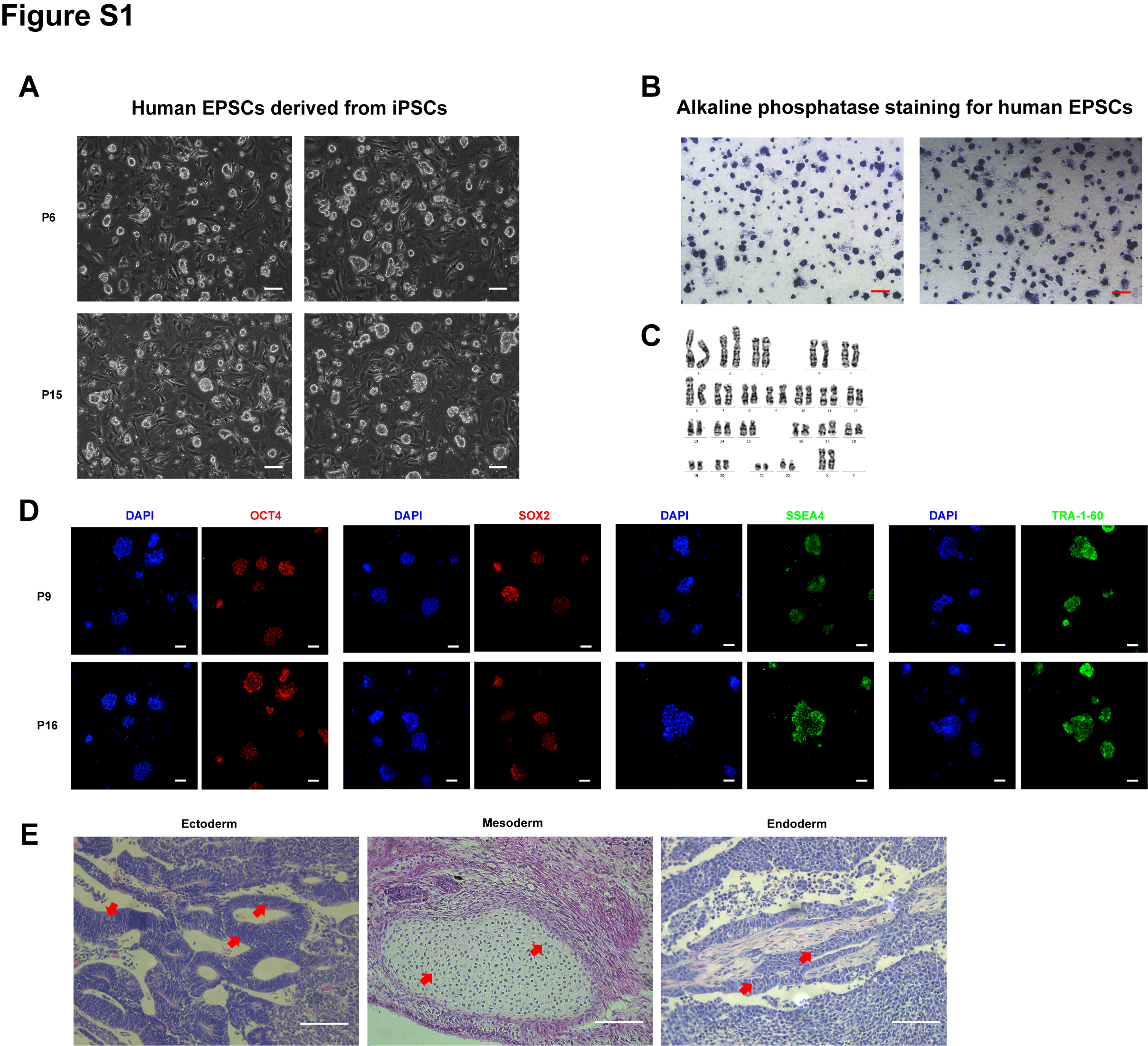


**Figure S1. Characterization of human EPSCs derived from human iPSCs**

(A) Representative images of human EPSCs induced from hiPSCs at P6 and P15.

(B) Representative images of human EPSCs detecting alkaline phosphatase activity. Scale bars indicate 100 μm.

(C) Representative karyotypes of human EPSCs at P10.

(D) Representative images of human EPSCs detecting OCT4, SOX2, SSEA4 and TRA-1-60 proteins by immunofluorescence. Scale bars indicate 100 μm.

(E) Representative images of human EPSC-derived teratoma showing differentiation of three germ layers by immunohistochemistry staining. Red arrows point to respiratory epithelial tissue in the endoderm, cartilage tissue in the mesoderm and neuroepithelial tissue in the ectoderm. Scale bars indicate 100 μm.


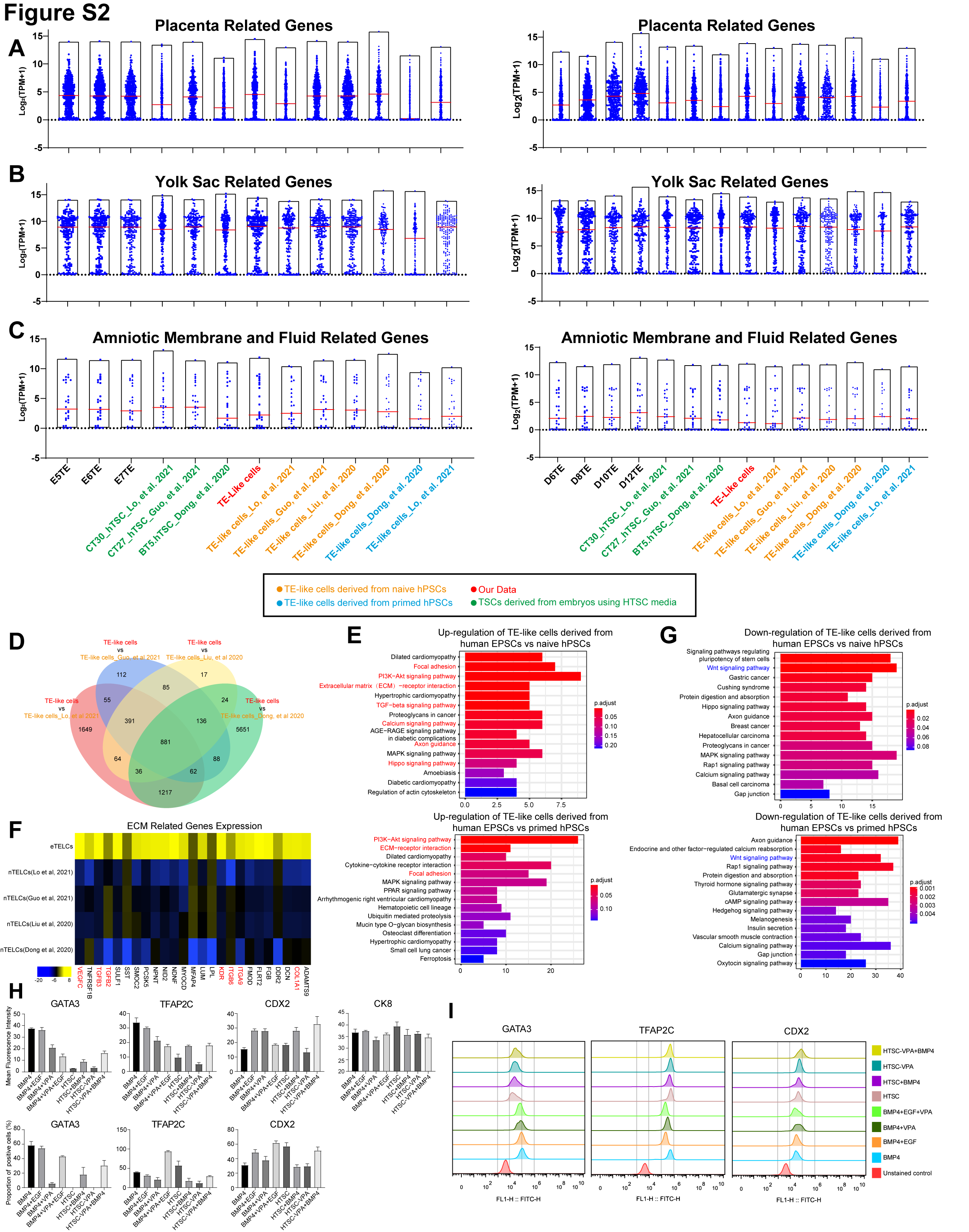


**Figure S2. TE-like cells derived from human EPSCs share a similar gene expression pattern with hTSCs, and those from naive hPSCs did not completely turn off naive-specific gene expression (related to Figure 2).**

(A) Expression of placenta-related genes in TE-like cells derived from human EPSCs, naive hPSCs, primed hPSCs, and hTSCs compared with single-cell RNA-seq data from human early embryos in vivo (left panel, ^1^) and in vitro (right panel, ^2^). The expression levels of genes are represented using the average of replicates or single cells’ Log2(TPM+1). Each blue dot represents one gene. Red lines in each column represent the median expression among all genes.

(B) Expression of yolk sac-related genes in the same samples in (A) with the same analysis.

(C) Expression of amniotic membrane- or fluid-related genes in the same samples in (A) with the same analysis.

(D) Venn diagram showing overlap of all genes with upregulated expression with comparison between TE-like cells derived from human EPSCs and naive hPSCs.

(E) Using genes with upregulated expression in TE-like cells derived from human EPSCs with an expression fold change greater than 2 compared to those from naive hPSCs (upper panel) and primed hPSCs (bottom panel), KEGG analysis was performed by clusterProfiler in R. The X-axis represents the number of genes, and the Y-axis represents the correlation between differentially expressed genes and various signaling pathways or biological processes.

(F) Heatmap of the expression patterns of selected ECM-related genes in TE-like cells derived from human EPSCs and naive hPSCs.

(G) Using genes with downregulated expression in TE-like cells derived from human EPSCs with an expression fold change of more than 2 compared to those from naive hPSCs (upper panel) and primed hPSCs (bottom panel), KEGG analysis was performed as shown in Figure S2E.

(H) Quantification of mean fluorescence intensity using the immunofluorescence staining images detecting TE-specific markers as shown in Figure 3C (upper panel). Fluorescence positive cell number counting using the same images (bottom panel).

(I) Flow cytometric analysis of TE lineage markers expression in TE-like cells induced by different groups.

**
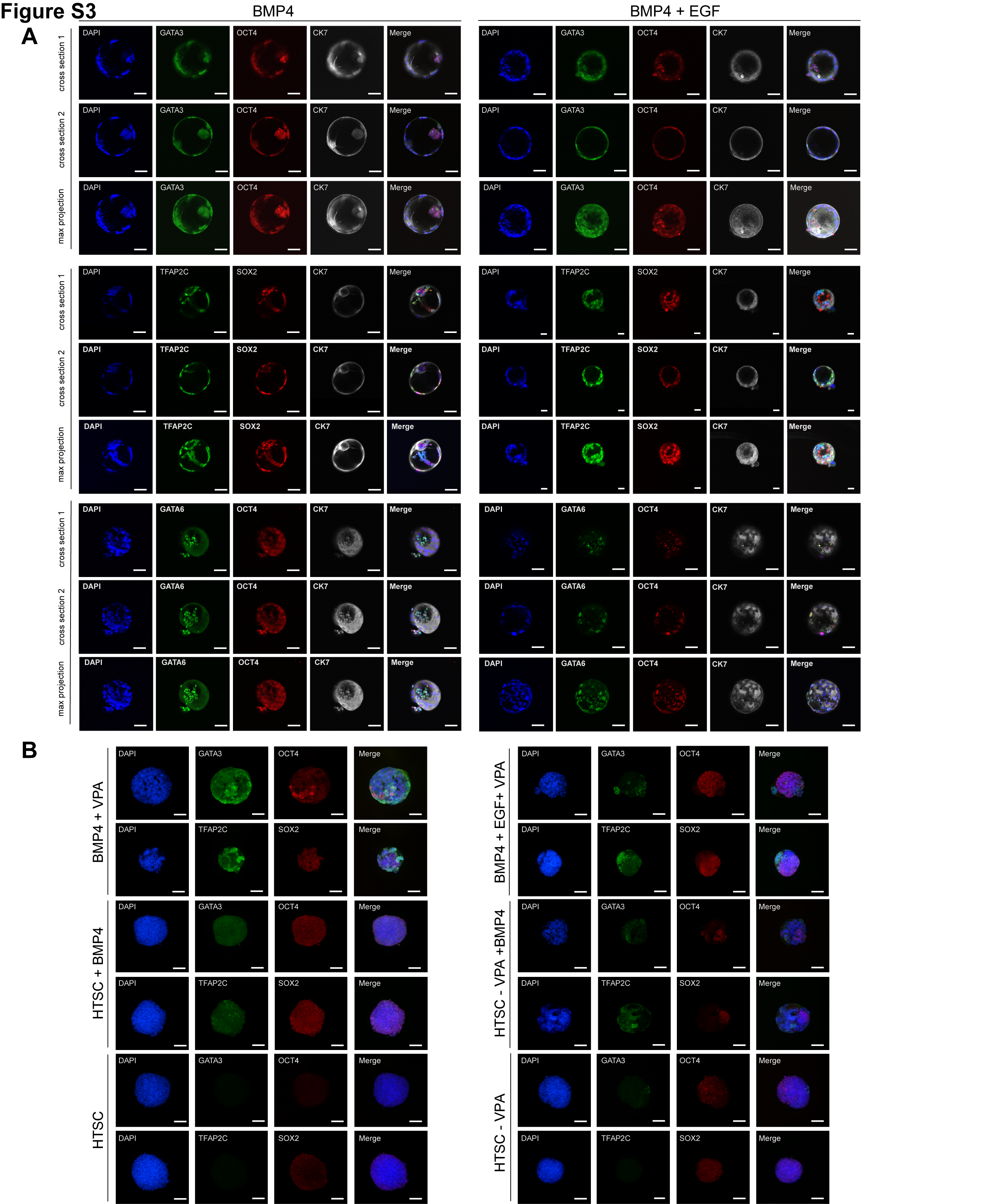
**

**Figure S3. Human blastoid reconstruction using TE-like cells derived from human EPSCs induced by multiple small molecule combinations, related to Figure 4.**

(A) Z axis scan of representative images of blastoids reconstructed from TE-like cells induced by BMP4 and BMP4+EGF, detecting the expression of TE lineage-specific markers (*GATA3, TFAP2C, CK7*), primitive endoderm maker (GATA6) and pluripotent markers (*OCT4, SOX2*) by immunofluorescence staining. All images were taken at Day 6 during induction. Scale bars indicate 100 μm.

(B) Representative images of blastoids using TE-like cells derived from multiple induction conditions, detecting the expression of TE-specific markers (*GATA3, TFAP2C*) and pluripotent markers (*OCT4, SOX2*) by immunofluorescence staining. All images were taken at Day 6 during induction. Scale bars indicate 100 μm.


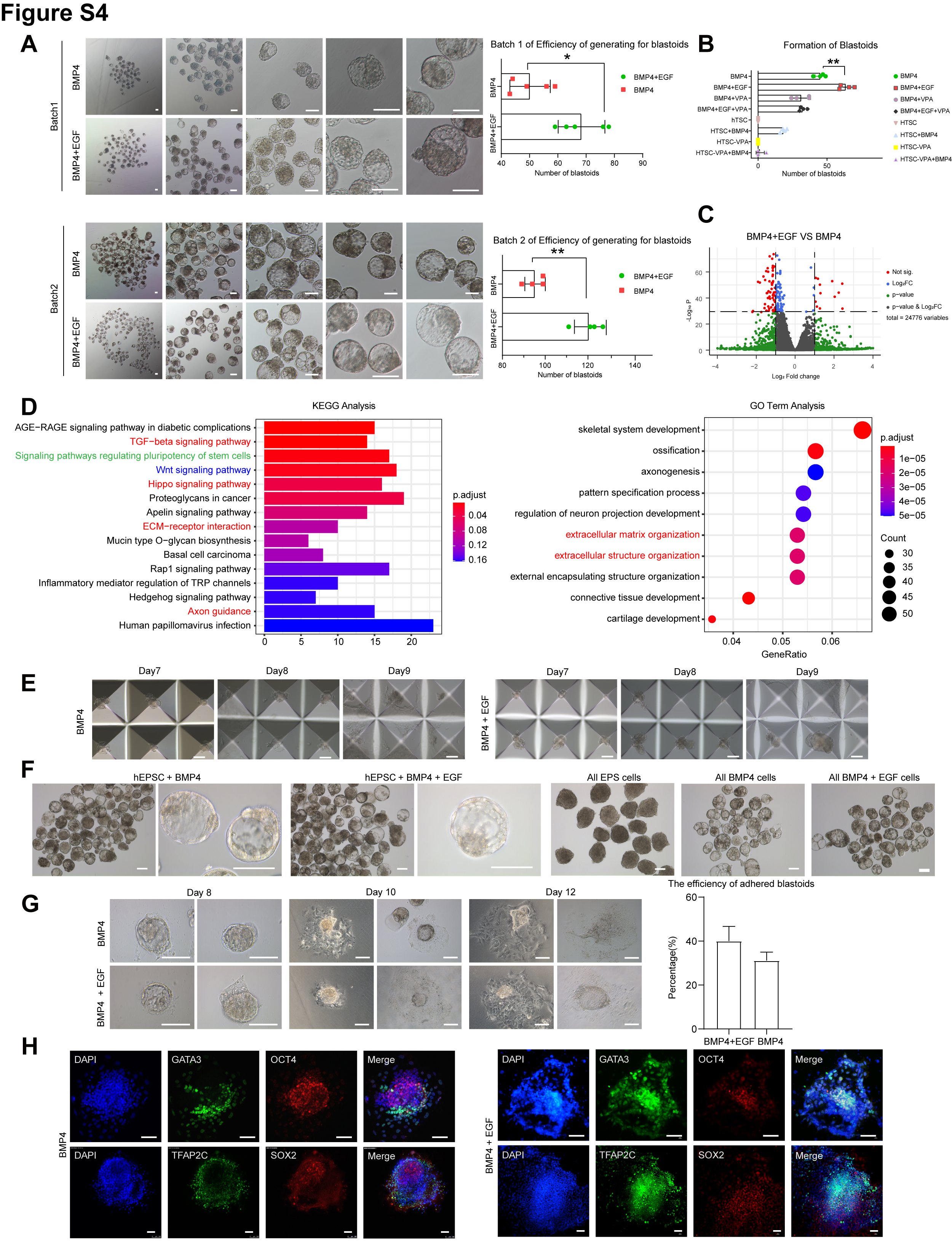


**Figure S4. Human blastoid construction is improved by further enhancement of TE-specific signal pathways by adding EGF, related to Figure 4.**

(A) Left panels: representative images of human blastoids using TE-like cells induced by BMP4 and BMP4+EGF at Day 6 within 2 batches. Batch 1 represents the number of human blastoids in each well, and batch 2 represents the total number of two wells. Scale bars indicate 100 μm. Right panels: quantitative statistics of the number of blastoids. **P* < 0.05, ***P*< 0.01.

(B) Histogram shows the numbers of human blastoids using TE-like cells from multiple induction conditions.

(C) The DEGs between the TE-like cells induced by BMP4 and BMP4+EGF. Samples were defined with uncorrected P values smaller than 0.01 and log_2_(fold change) larger than 0.25 (log2FC > 0.25) in one group.

(D) Using genes with upregulated expression in TE-like cells induced by BMP4+EGF with expression fold changes more than 2 compared to TE-like cells induced by BMP4, KEGG analysis (left panel) and GO term (right panel) were both performed by clusterProfiler in R. X-axis represents the number of genes, and Y-axis represents the correlation between differentially expressed genes and various signal pathways or biological processes.

(E) Representative images of blastoids using TE-like cells induced by BMP4 and BMP4+EGF at Day 7, Day 8 and Day 9. Scale bars indicate 100 μm.

(F) Representative images of blastoid-induced BMP4 and BMP4+EGF and generated blastoids under different combinations. Each symbol represents the number of blastoids in each well. Scale bars indicate 100 μm.

(G) Left panel: representative images of blastoids using TE-like cells induced by BMP4 and BMP4+EGF at Day 8, Day 10 and Day 12 in the extended culture system in vitro. Scale bars indicate 100 μm. Right panel: histogram shows the numbers of blastoids adhered.

(H) Representative images of blastoids reconstructed from TE-like cells induced by BMP4 and BMP4+EGF, detecting the expression of TE-specific markers (*GATA3, TFAP2C*) and pluripotent markers (*OCT4, SOX2*) by immunofluorescence staining. All images were taken at Day 10 during induction. Scale bars indicate 100 μm.


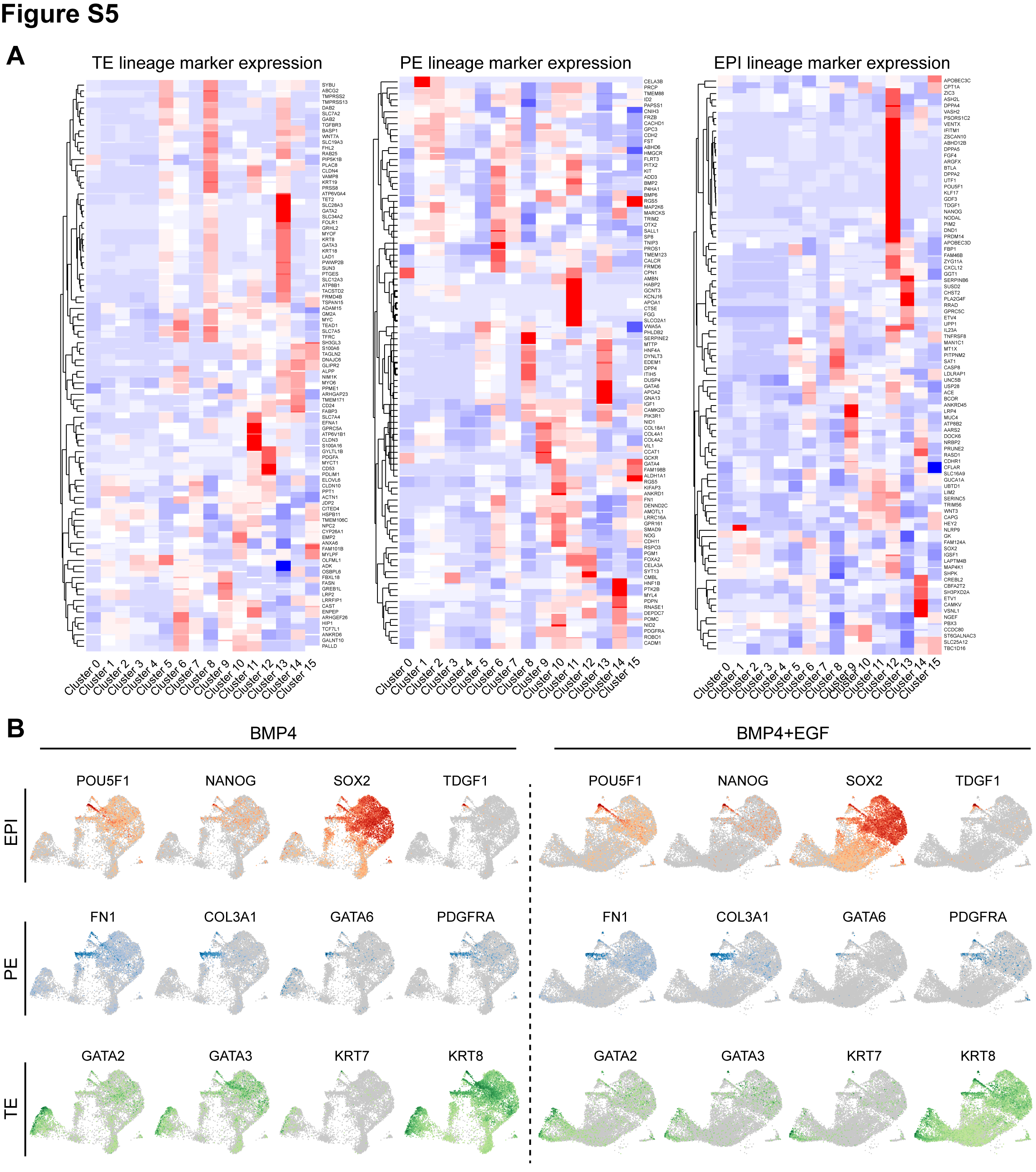


**Figure S5. Gene expression patterns of three lineages of blastoids generated from human EPSCs, related to Figure 5**

(A) Heatmap showing lineage gene expression of TE (left panel), PE (middle panel) and EPI (right panel) cell populations using integrated datasets of BMP4 and BMP4+EGF blastoids.

(B) UMAP plots showing EPI, PE and TE lineage-specific gene expression using integrated datasets of BMP4 and BMP4+EGF blastoids.
